# In their own words: deception detection by victims and near victims of fraud

**DOI:** 10.3389/fpsyg.2023.1135369

**Published:** 2023-05-12

**Authors:** Marianne Junger, Luka Koning, Pieter Hartel, Bernard Veldkamp

**Affiliations:** ^1^Industrial Engineering and Business Information Systems (IEBIS), Faculty of Behavioural, Management and Social Sciences (BMS), University of Twente, Enschede, Netherlands; ^2^Department of Services, Cybersecurity and Safety, Faculty of Electrical Engineering, Mathematics and Computer Science, University of Twente, Enschede, Netherlands; ^3^Department of Cognition, Data and Education (CODE) Faculty of Behavioural, Management and Social Sciences (BMS), University of Twente, Enschede, Netherlands

**Keywords:** online fraud, fraud victimization, crime victimization, cybercrime, human factors, deception-detection

## Abstract

**Aim:**

Research on deception detection has usually been executed in experimental settings in the laboratory. In contrast, the present research investigates deception detection by actual victims and near victims of fraud, as reported in their own words.

**Materials and methods:**

Our study is based on a nationally representative survey of 11 types of (mostly) online fraud victimization (*N* = 2,864). We used qualitative information from actual victims and near victims on why they didn’t fall for the fraud, or how, in hindsight, it could have been prevented.

**Results:**

The main detection strategies mentioned by near victims (*N* = 958) were 1) fraud knowledge (69%): these near victims clearly recognized fraud. Other strategies related to fraud knowledge were: noticing mistakes (27.9%), rules and principles about safe conduct (11.7%), and personal knowledge (7.1%). A second type of strategy was distrust (26.1%). A third strategy was ‘wise through experience’ (1.6%). Finally, a limited number of respondents (7.8%) searched for additional information: they contacted other people (5.5%), sought information online (4%), contacted the fraudster (2.9%), contacted their bank or credit card company (2.2%), or contacted the police (0.2%). Using knowledge as a strategy decreases the probability of victimization by a factor of 0.43. In contrast, all other strategies increased the likelihood of victimization by a factor of 1.6 or more. Strategies generally were uncorrelated, several strategies differed by type of fraud. About 40% of the actual victims (*N* = 243) believed that their victimization might have been prevented by: 1) seeking information (25.2%), 2) paying more attention (18.9%), 3) a third party doing something (16.2%), 4) following safety rules or principles, like using a safer way of paying or trading (14.4%), or by 5) ‘simply not going along with it’ (10.8%). Most of these strategies were associated with a higher, not lower, likelihood of victimization.

**Conclusion:**

Clearly, knowledge of fraud is the best strategy to avoid fraud victimization. Therefore, a more proactive approach is needed to inform the public about fraud and attackers’ modus operandi, so that potential victims already have knowledge of fraud upon encountering it. Just providing information online will not suffice to protect online users.

## 1. Introduction

Fraud can be defined as ‘*crime* […] *targeted against individuals* [that use] *deception for the purpose of obtaining illegal financial gain.* [It] *involves the misrepresentation of facts and the deliberate intent to deceive with the promise of goods, services, or other financial benefits that in fact do not exist or that were never intended to be provided* (Titus et al., 1995). In practice, the ‘fraud’ label covers a broad range of activities (for a summary, see Levi and Burrows, 2008) such as telemarketing fraud, fraud involving financial services, insurance coverage, investment or business schemes and fake charities (Titus et al., 1995). Fraud is mostly online, today (Beals et al., 2015; DeLiema et al., 2017; Button and Cross, 2017a). Cybercrime consists to a large extent of ‘online fraud’.

Fraud is a growing problem. In the Western world, registered crime and victimization have been declining since the late 1990s (Blumstein and Wallman, 2005; Farrell, 2013; Button et al., 2014; Hopkins, 2016; De Jong, 2018; Levi and Doig, 2020). However, in stark contrast, fraud increased relatively strongly in many Western countries during the past two decades. Fraud statistics are showing an alarming increase, with new peaks in the United States (Finklea, 2014; Javelin, 2014), in the United Kingdom (Financial Fraud Action UK, 2017; Button and Cross, 2017b) and elsewhere in Europe (Statistics Netherlands, 2018; Junger et al., 2020; Kemp et al., 2020). The present study investigates the strategies used by victims and near victims to recognize fraud, and can inform us on how to better protect consumers and online users.

Similar to Titus et al. (1995), most scholars considered fraud a form of deception (Baesens et al., 2015; Van Vlasselaer et al., 2015; Oxford Dictionaries, 2018). In line with this, it is not surprising that deception has been investigated with some regularity in fraud research (Stajano and Wilson, 2011). But deception has also been studied in the study of social engineering (Mouton et al., 2014; Bullée, 2017; Steinmetz, 2020; Bullée and Junger, 2020b; Steinmetz et al., 2021; Washo, 2021), in marketing (Goldstein et al., 2008), and in psychology (Grazioli and Wang, 2001; DePaulo et al., 2003; Hancock and Gonzales, 2013; Burgoon and Buller, 2015; Levine, 2019, for an overview, we refer to Docan-Morgan, 2019). By investigating into deception, we can gain more insight into successful strategies to prevent victimization.

In the review below, we will focus on the psychological literature on deception that presents some concepts that are applicable to the present study, and we present a brief review of the relevant fraud literature. These two bodies of research are to some extent complementary. The psychological literature has mostly focused on the receivers of deceptive communication: how do people recognize deception? In contrast, the fraud literature typically investigated (a) the senders, in the present case the fraudsters: how do they manage to deceive, and (b) the judges/receivers’ characteristics, in the present case the victims: who is most likely to be defrauded? Accordingly, little information is available in the fraud literature on how fraud victims recognize deception, more specifically, how do they deal with online offers for products, services or unsolicited emails, or why they fall for scams (Button et al., 2014). Below we start with a summary of some of the main findings in both the psychology of deception detection and on fraud detection.

### 1.1. Psychological research on deception

A large body of psychological research investigated deception detection in human interactions (Vrij, 2019; Masip Pallejá et al., 2021), and has been summarized in several publications (Aamodt and Custer, 2006; Bond and DePaulo, 2006; Hartwig and Bond, 2011, 2014; DePaulo and Bond, 2012; Evans et al., 2012; Hauch et al., 2012, 2014; Suchotzki et al., 2017; Vrij et al., 2017, 2019; Levine, 2019; Verschuere et al., 2021).

People are not very good at recognizing deception in an experimental setting: in their meta-analysis, Bond and DePaulo (2006) analyzed 206 studies with a total of 24,483 experimental ‘judges’. The experimental judges had to discriminate lies from truths in real-time without any aid or training. In these circumstances, people achieved an average of 54% correct lie–truth judgments, correctly classifying 47% of lies as deceptive and 61% of truths as nondeceptive (Bond and DePaulo, 2006).

The Truth-Bias, or ‘veracity effect’, as it was labelled by Bond and DePaulo (2006) is part of the explanation for this apparent lack of ability in recognizing deception. People generally start with the presumption of truth (Burgoon and Levine, 2010; Street, 2015; Street et al., 2019; Armstrong et al., 2021; Masip Pallejá et al., 2021; Levine, 2022). This seems sensible, as several studies demonstrated that most people tell the truth, most of the time (Serota et al., 2010; Levine, 2019). Accordingly, an observation or a noticeable cue is necessary to trigger suspicion. Research tried to discover why people are not very good at deception detection and whether they can improve, for instance through training. A number of possible factors could be the following (see Burgoon and Buller, 2015 for a review).

Researchers studied *deceiver social skills.* A small number of deceivers are convincing liars and accordingly are hard to detect (Burgoon and Buller, 2015), while some deceivers may be relatively easier to detect (Evans et al., 2017). Research also investigated *Judge’s detection skills.* Generally, those who are asked to detect a lie, ‘judges’, perform equally well – or poorly (Bond and DePaulo, 2008). There are hardly any differences in detection skills by age, education and experience (Vrij and Mann, 2001; Levine, 2019). *Context and amount of exposure* also matters. When judges of interpersonal communication receive more context or background knowledge, they perform better at detecting deception (Burgoon and Buller, 2015). A lot of studies focused on the *message and the cues to deception.* Research examined verbal aspects of an account, such as the level of detail in an account, vocal tension, logical structure of a story, negativity in statements, and visual factors, such as nervousness or fidgeting (DePaulo et al., 2003; Vrij et al., 2019). However, several studies concluded that most cues to deception are weak and not very useful to detect deception (DePaulo et al., 2003; Hartwig and Bond, 2011; Luke, 2019).

### 1.2. Deception detection in real life

Research on deception detection has been criticized. Its main problem, according to Park et al. (2002) is a lack of external validity. Most research relied on laboratory experiments with senders who lied or told the truth and judges who had to figure out if they lied. Judges and senders do not know each other, there is no interaction and no possibility to ask questions or fact-checking. The senders do what they are told to do, and the stakes are minor. What is left is a focus on verbal and non-verbal behavior that precludes other sources of information. All this is far away from what happens in real life, and what may lead people in the real world to detect a lie (Park et al., 2002). Consequently, Park et al. (2002) set out to ask people whether they recalled having been lied to and how they discovered that. They find that, in real life, lies are discovered mainly by third-party information (32%), physical evidence (18%), an unsolicited confession (8%) or some combination. Only 14.9% are discovered at the moment they are told, and most lies are discovered relatively late (Park et al., 2002). Several follow-up studies confirmed the importance of fact-checking and evidence (Blair et al., 2010; Masip and Herrero, 2015; Novotny et al., 2018; Levine and Daiku, 2019; Masip Pallejá et al., 2021).

Timing is different in real-life in comparison with laboratory experiments. In experimental studies, judges are asked to detect (or not) the lie on the spot. But because fact-checking is usually something that cannot be done immediately, in real life most lies are discovered sometime after they were told. Park et al. (2002) reported that only 14.9% of the lies were detected at the time they were told, 80.9% of the lies were discovered more than an hour after they were told, 60.3% were detected more than a day later, and 39.7% were uncovered more than a week later. Several studies replicated this finding (Masip and Herrero, 2015; Levine, 2019; Masip Pallejá et al., 2021). Also, many lies are discovered unexpectedly (Masip Pallejá et al., 2021). These findings emphasize the fact that verbal and non-verbal behavior do not play a significant role in lie detection (Masip, 2017). An interesting question is whether this applies to fraud, online or offline.

### 1.3. The importance of context

As mentioned above, most people start interpersonal communication with a Truth-Bias. Street (2015) stated that the Truth-Bias can change to become a lie-bias, depending on the context: ‘*According to Adaptive Decision Strategies in Lie Detection (ALIED), the presence and direction of the bias is all a matter of context: Relying on context-general information (“most people will lie/tell the truth”) can be a useful aid to making an informed judgment in the absence of more precise information*’. ‘Context-general information’ tells us how likely it is that one may encounter a lie in a specific situation. In uncertain situations, people rely on generalized rules based on their knowledge of the situation (Street, 2015).

Besides context-general information, people can use ‘individuating information’. In Street’s model, ‘individuating information’ is information about a single specific statement, rather than about statements in general. Because of its specificity, individuating information usually has poor diagnostic value (Street, 2015; Street et al., 2016). For instance, if ‘I went home after class’ was a lie, this usually does not help much in terms of judging other statements of people. This specific information could allow almost perfect deception detection in certain conditions. For instance, one condition is that people need to pay attention to individuating cues (Street, 2015). ‘*raters trade-off individuating information with more context-general information so that as the individuating information becomes less diagnostic there is a greater influence of context.’* According to Street (2015). Individuating information is to be preferred, but when that is absent, context-general information needs to be used.

In line with ALIED, several researchers emphasized the importance of knowledge of context in real-life deception detection (Street, 2015; Street et al., 2019; Masip Pallejá et al., 2021). Based on his new theoretical account, (Street, 2015) concludes that individuals will make use of their knowledge of the world to make informed judgments about the truth.

### 1.4. Online communication

Today, it is important to distinguish between communication that occurs offline and online. Online fraud differs from deceit in interpersonal communication: it can, but it does not require personal interaction. Sometimes, online users must evaluate a possibly malicious website, an email, a WhatsApp message, or a text message. But it also consists of a phone call from someone posing as a help desk asking you for personally identifiable information or to log into your computer or transfer money to another bank account and there is an interaction with a fraudster.

Online users often have problems in identifying deception, similar to those involved in offline interpersonal communication (Williams et al., 2017; Norris et al., 2019): users have difficulties in recognizing phishing emails (Egelman et al., 2008), phishing websites (Downs et al., 2006; Purkait, 2012), fake advertisements and malicious web shops (Grazioli and Wang, 2001; Grazioli, 2004), or spoofed websites (Dhamija et al., 2006; Sheng et al., 2007; Lin et al., 2011).

### 1.5. Fraud research and the role of various forms of knowledge

Similar to research in offline interpersonal communication, research on online fraud tried to get a better grip on what happens when users are confronted with online fraud.

As mentioned above, a lot of fraud research has been focused on deceiver skills (Manky, 2013; Oest et al., 2018, Hyslip and Holt, 2019), fraudulent messages and persuasion techniques (Langenderfer and Shimp, 2001; Lea et al., 2009; Dolan et al., 2012; Button et al., 2014). Also, various studies investigated user’s socio-demographic characteristics (Anderson and Agarwa, 2010; DeLiema et al., 2017; Bullée and Junger, 2020b) and personality (Holtfreter et al., 2008; Wilsem, 2011; Fernández-Alemán et al., 2013; Pratt et al., 2014; Holt et al., 2018, 2020; Mesch and Dodel, 2018).

Below we focus solely on the impact of fraud knowledge and on ‘cross-situational’ cues, for reasons of space. In an online setting, knowledge can be important just as it is in offline interpersonal communication. Research reported that many users have insufficient knowledge and lack strategies to identify indicators of online fraud (Grazioli and Wang, 2001; Hong, 2012; Purkait, 2012; Acquisti et al., 2015). They do not know the methods fraudsters use to execute their fraud (Kritzinger and von Solms, 2010; Kritzinger and von Solms, 2013). The importance of knowledge is underscored by the fact that training improves online deception detection (Kumaraguru et al., 2010; Purkait et al., 2014). A recent meta-analysis reported some highly effective training methods which achieve a Standardized Mean Difference of 1 or more, which is unusually high (Bullée and Junger, 2020a).

Some studies, however, reported no relationship between fraud knowledge (knowledge about fraud/phishing) and unsafe online behavior; in these studies, the authors focused mostly on knowledge and practicing safe online behaviors (Holt et al., 2018; Leukfeldt et al., 2018; Van’t Hoff-De Goede et al., 2019).

A different look at knowledge impact was presented by Lea et al. (2009). These authors stated that the more knowledge near victims have about a specific field, the more they feel competent and, consequently, overestimate their abilities to take good decisions (Lea et al., 2009). For instance, victims of investment fraud have more knowledge in finance than non-victims. Lea et al. (2009) suggested that knowledge leads to ‘overconfidence’ which leads to biases in decision making for instance because it makes judges more selective in their information search (Anderson, 2016).

These different approaches to knowledge underscore the importance to distinguish between these two different types of knowledge: knowing and practicing safe behavior is something different from recognizing a malicious URL. Similarly, Lea et al. (2009) refer to ‘field knowledge’, which could be, for example, knowledge about the financial world. Later in this study, we write about ‘fraud knowledge’, which is knowledge about fraud, such as knowledge about investment scams; and this knowledge does not have to be related to knowledge of the financial world as such.

A problem with deception detection and fraud knowledge, is that it is hard to find cross-situational cues, that is, cues that would work for many or possibly all forms of deception or fraud (Burgoon and Levine, 2010; Burgoon and Buller, 2015). As fraud comes in uncountable varieties, we believe this is certainly true for fraud (Purkait, 2012; Button et al., 2014). For instance, a cross-situational cue could be ‘typos’ in an email. But phishing emails have improved their style and fraud is also executed more and more *via* telephone calls or text messages, so these ‘handy’ cues do not always work well in practice (NCSC, 2022).

In sum, there is a large body of research on deception detection based on laboratory experiments and on real-life deception detection. There is some research on fraud and fraud victims. But, according to Lea et al. (2009), ‘*The available research on scams is, for the most part, fragmented, descriptive, and non-psychological’*. Moreover, only a limited number of studies asked victims who experienced an attempted fraud to report what, in their own words, helped to avoid victimization. Those that did generally used relatively small samples and focused on the persuasive messages, not on what helped the victims to detect the fraud. Also, Fischer et al. (2013) combined victims and near victims who were confronted with a fraud attempt, which may blur differences between both categories.

The present study focuses on the victim, not the fraudulent message. It examines two main questions: (a) what preventive strategies are used by near victims, who experienced a scam attempt, to avoid falling for the fraud, and (b) what strategies, according to the victims, could have prevented them from falling victim to fraud? Our study will compare the preventive strategies of victims and non-victims, and it will examine whether strategies are interrelated and whether specific strategies are used for the different forms of fraud. It is based on a random sample of the Dutch population and investigated the prevalence of fraud. Respondents were given the opportunity to describe, in their own words, the fraud and the reasons for falling or not for the fraud.

## 2. Materials and methods

### 2.1. Fraud victimization survey sample

The current study analyszs data from a fraud victimization study reported upon by Junger et al. (2022). Data were collected using an online Dutch questionnaire that was administered *via* the LISS panel [Longitudinal Internet Studies for the Social Sciences panel (Centerdata, 2021)]. The LISS panel (managed by CentERdata, related to Tilburg University) is an online panel consisting of approximately 5,000 households, roughly 7,500 individuals in the Netherlands. Participating households were recruited by means of a random sample from the population register of Statistics Netherlands. If households do not have a computer and/or internet connection, they are provided with one or both in order to participate (Centerdata, 2021). This recruitment method provides very good representativeness of the population (De Vos, 2010; Scherpenzeel and Bethlehem, 2011; Brüggen et al., 2016; Eckman, 2016).

Data collection took place early 2021, January 11 to February 2, and asked respondents about fraud victimization in Statistics Netherlands (2020). 3,623 randomly selected LISS panel members were invited to participate in the fraud victimization study, of which 2,920 started the questionnaire. After the selection on completed questionnaires, 2,873 respondents remained. The removal of 9 respondents who gave unreliable answers resulted in a final sample of 2,864 respondents. The response rate was 79%.

Males constitute 44.9% of the final sample, and females 55.1%. The mean age of the sample is 53, with a standard deviation of 18.4, the minimum age was 16 and the maximum was 95. The average imputed household net monthly income was € 3,407, with a standard deviation of 3,401, the minimum was 0 and the maximum was € 147,416. Educational level was defined as the highest educational level, regardless of diploma. 2.3% of the respondents only had followed elementary school, 17.5% followed pre-vocational secondary education, 7.3% followed high school, 22.2% followed intermediate, vocational training, 30.6% followed higher vocational education, 18.4% followed university education, 1.3% did not follow any formal education.

A comparison of the present sample with the distribution within the Dutch population (Centerdata, 2021) shows that there were slightly too few men (−4.5%). The youngest age group (16–24) was also underrepresented (−5.1%) and the elderly (65 and older) were overrepresented (+8.6%). Finally, persons with higher vocational education were overrepresented (+6.1%) while persons with an intermediate vocational education were underrepresented (−4.4%).

### 2.2. Measures

The fraud victimization questionnaire was based on a pilot by DeLiema et al. (2017), which was conducted in the United States. This pilot used Titus et al. (1995)’ definition of fraud, as mentioned above. DeLiema et al. (2017)’ fraud taxonomy was used in a slightly adapted version, and it was expanded. One concept, ‘relationship fraud’ was split into dating fraud and friend-in-need fraud (such as ‘WhatsApp’ fraud). Furthermore, questions were added about identity fraud, based on the Security Monitor of Statistics Netherlands (2020), phishing, based on work by Näsi (2022) and spoofing, which includes ‘help desk fraud’. Table 1 shows schematically the fraud taxonomy.

**Table 1:** Fraud taxonomy.

Fraud category	How often did it happen that …
Investment fraud	… you invested your money because someone promised high or guaranteed returns, but the investment yielded much less or your money was not invested at all?
Purchase fraud	… You paid for a product or service that you never received or that was a scam?
Job fraud	… you paid to get a job that did not exist, a fake job vacancy that made you lose money or wasn’t as profitable as promised?
Prize fraud	… you paid to receive a prize, grant, inheritance, or lottery winnings that you never received?
Debt fraud	… you paid to pay off a debt that did not exist or for an account of something that you did not buy?
Charity fraud	… you donated money to a charitable organization or charity (for example on a crowdfunding website) that was (probably) fake?
Dating fraud	… you gave or lent money to someone who pretended to be in love with you?
Friend-in-need fraud (including ‘WhatsApp fraud’)	… you gave or lent money to someone who pretended to be a relative, friend, or acquaintance of yours?
Phishing	… you gave your username, password, or bank or credit card information to outsiders in response to email or website phishing.
Identity fraud	(in addition to the previous questions…) How often has someone made use of your personal data (e.g., name, bank details, social security number/social security number) without your intention, for financial gain, for example, to withdraw or transfer money, take out a loan, request official documents, buying products and/or services or taking out subscriptions?
Spoofing (including ‘helpdesk fraud’)	(in addition to the previous questions…) … you lost money because someone pretended to be someone else (e.g., an employee of your bank)?
Other types of fraud	(in addition to the previous questions…) How many times has something else happened where you paid money because someone misrepresented information, lied about information, or withheld information?

For each fraud category in Table 1, respondents indicated how often they were victimized in the past year (1 January to 31 December 2020) and in the past 5 years (1 January 2016 to 31 December 2020). Information on the past year was used in the present study.^1^ Respondents also indicated for each type of fraud whether they had experienced a fraud attempt and, if so, whether they responded to it. This was not asked in the case of identity fraud, because this form of fraud does not require contact between the perpetrator and the victim. Loss of money was required to be classified as a victim. An exemption was made in the case of phishing and identity fraud; victimization was possible for those forms if a respondent’s data had been stolen or abused. Next, respondents were asked additional questions about the most important fraud victimization and about the most important fraud attempt. Finally, respondents were asked some background questions.

### 2.3. Current analysis

The current study analyses open-text answers about the most important fraud victimization and the most important fraud attempt. For both the attempt and the actual victimization, respondents were first asked to describe, in their own words, what happened. Questions were then also asked about how victimization was prevented or could have been prevented. Again, we asked respondents to describe this in their own words. This was done because we did not want to suggest answers but wanted to register respondents’ own accounts (Züll, 2016). Similar approaches were used in other security research dealing with user information (Lea et al., 2009; Levine and Daiku, 2019; Breen et al., 2022).

For both victimization and the attempts, the coding of the qualitative answers was done in an iterative process, as described by Züll (2016). Beforehand, no specific codes were expected, and no previous research could be found to guide the coding process.

For the fraud attempts, a codebook was developed by the first author and the third author while reading the answers; it was checked by the second author and, after discussions, a new version of the codebook was established, which was used to code all answers by the first and the second author. Differences in coding were discussed, after which the final version of the codebook was established’. A similar procedure was followed for the coding of the actual fraud victimization incidents. The codebook was developed by the second author and checked by the first author and the final coding was performed by the second author. In developing the code book of attempts, we took possible preventive actions and potential policy measures into account, as will be explained below.^2^

For attempts, kappa’s ranged from 0.96 to 1, and percentages of agreement ranged from 93 to 100%; for victims, the agreement was 100%.

*Language issues.* As mentioned above, the questionnaire was administered in Dutch, and all respondents typed their answers in Dutch, with one exception, who wrote English but whose answers were not used for quotes. Accordingly, the quotes we add below have been translated by us, by using Google Translate and verifying the translation for the correct meaning.

#### 2.3.1. Attempted victims

Respondents had to think of their most memorable fraud attempt in, 2020 and were asked to describe, in their own words, what had happened. They were also asked why they thought they did not lose money: (a) ‘*Why did not you pay?’*, or for those with identity fraud as the memorable attempt the question (b) ‘*Why did not you lose money’*. Both variables (a and b) were combined into one. Respondents were also asked about if they noticed something that was not right during the fraud; if they answered that they did, a request followed to describe what they had noticed. The fraud attempt description, the answer on why respondents did not lose or pay money, and the answer on what respondents noticed that was not right were bundled and coded as one. This was done because respondents sometimes already mentioned what they had noticed as wrong or fraudulent in the description of the attempt, and the context given by the description was sometimes needed to understand the other answers. Thirteen codes were developed and assigned for strategies to detect and resist fraud attempts.

(1) Fraud knowledge. The respondents indicate that they recognized the fraud attempt, based on knowledge about fraud. Knowledge was also coded when indicators of fraud were described, such as ‘typos’, ‘poor writing style’, other stylistic errors, or a ‘foreign accent’. Usually, several of these indicators were mentioned in combination. Examples are: ‘[the] *email address was incorrect’, ‘the accent, the poor language skills, and the so-called big company names. As [I already] said, I have not said anything and imposed’,* and *‘yes, I follow the current fraud trends!’.*
(2) Distrust. The respondent recognizes the fraud attempt, based on a feeling that something was not right, often without further specification: something seems unreliable, unclear, strange, or weird. Examples are: *‘was too insecure’, ‘was very suspicious’, ‘[I] do not trust these emails’,* and *‘emotionally it did not make sense’.*
(3) Rules and principles. This code was assigned when respondents mention that they have personal rules and principles about being careful and or (not) doing certain things. These rules and principles help them to avoid falling for fraud. Examples are: *‘I am always alert’, ‘I would never send a debit card and code’,* and *‘I always check the email address before opening anything’.*
(4) Independent information seeking. This code was assigned when certain information was missing and/or when respondents independently searched for more information, leading them to recognize the fraud. This includes respondents searching for information online. Examples are: *“I have verified the accuracy and found that this was a fraud’, ‘Wrong water company. Looked up on the Internet’* and *‘I googled it and found the same texts on forums where people were warned’.*
(5) Mistakes. Respondents note facts that are incorrect or do not match the respondent’s situation. For instance, he/she has no children, he/she does not bank with that specific bank. These mistakes refer to anything that the offender could figure out from the internet. Examples are: ‘*I do not have children’, ‘It was clear that the facts do not add up’,* and *‘I do not bank with that bank’*. From a policy point of view, we note that, with a little effort, the fraudsters could improve their messages in such a way that the near victim no longer recognizes the fraud attempt and avoids these mistakes.
(6) Personal knowledge and private context. This code was assigned when a respondent recognized the fraud attempt based on knowledge of their family context or connections or any information that is not available to the fraudster, not available online but personal and private knowledge of the victim. In these cases, a fraudster is impersonating a family member, but the respondent knows this family member would never act the way that the supposed family member is acting. Examples are: ‘[I recognized this] *directly. My father would never do that’, ‘weird app, my kids would never communicate anything like this’, ‘my daughter would never ask that’,* and *‘would be weird if this person asked me to’*. This was done with an eye on possible policy implications. This type of mistakes cannot be easily corrected even if the attackers would be able to collect much more information on a potential victim.
(7) Contact with the bank or the credit card company. Some respondents mentioned that they had contact with their bank or credit card company, for information or about blocking transactions.
(8) Contact with online shops and trading platforms. Some respondents contacted the online shop involved in an email or a transaction. One respondent noted: *‘Because, on the advice of the employee, I changed my password of my e-mail account and for bol.com. (I was very disappointed with myself for opening the email)’*.
(9) Contact with others. Some respondents sought contact with others about the fraud, or occasionally were contacted by others about the fraud. This includes respondents discussing the event with relatives, to gauge whether it may be fraud or not. Examples are: ‘*Checked by calling her (WhatsApp)’, ‘In conversations with friends and relatives, it turned out that several people had received an assessment about a fictitious overdue tax amount’,* and *‘Check via the authority, after which it turned out that it was indeed phishing’.*
(10) Wisdom through experience. Respondents also mentioned they were victimized in the past or had bad previous experiences and this was the reason they did not fall for the current attempt. For instance, respondents mentioned: ‘[I] *recognized the trick. Had happened before and then I fell for it’* and *‘Didn’t trust it from previous experience’.*
(11) Contact with police. Some respondents mentioned that they called the police to check the content of the fraud. Two examples are: *‘On the advice of the police I ignored the invoice and I never heard anything about it again’,* and *‘I found out through the police that it was a scam’.*
(12) Check with the attacker. Sometimes respondents contacted the attacker, mostly to check things. For instance*, ‘I wanted more information’, ‘he was having a hard time answering questions from my side’, ‘When I asked questions, I got strange answers and unclear prognosis’* (in the case of investment fraud) and *‘Information requested by me was not immediately given’.*
(13) Something else. Various answers were given that were rarer and/or not easy to classify.

An additional code was assigned for respondents that mentioned explicitly that they recognized the fraud attempt immediately or emphasized speed in recognition and action. This is not a strategy but is informative and was analyzed as well.

Codes for attempts were not mutually exclusive. If respondents mentioned multiple reasons, multiple codes were assigned.

#### 2.3.2. Actual victims

Respondents were first asked if they noticed something beforehand and if they thought someone could have prevented the experience, and who (a wide range of actors were listed, including the participant themselves and an ‘other’ option). If any actor was chosen respondents were then asked questions on prevention, among which, how, they believed, the victimization could have been prevented.

The description of the fraud was combined with the answer to a question about how respondents thought the experience could have been prevented. Ten codes were developed and assigned for strategies that could have prevented fraud incidents.

(1) Simply not doing it. Respondents mentioned that they just should not have done what they did, without specifying further, for example: ‘*not doing what I did*’.
(2) Distrust. Respondents mentioned that they should have been more distrusting and/or less gullible. Examples are: ‘*not trusting everything’, ‘not being gullible’.*
(3) Being more alert. Respondents reported that they should have paid more attention to signals that were present which indicated fraud (without searching for more information), for instance, ‘*paying more attention*’.
(4) Thinking better. Respondents believed they should have thought better before taking an action. This included taking more time before acting, for example: ‘*thinking carefully first*’.
(5) Independent information seeking. Some respondents indicated they should have looked for more information by themselves. This includes asking the fraudster for more information, for example: ‘*asking for more information*’.
(6) Contact with others. Respondents replied that they should have contacted others (not the fraudster). This could be a third party or the person/organization concerned that the fraudster is posing as. Examples are *‘If my parents or my brother had explained to me what was going on’, ‘That my father had called me again on my telephone number’* and *‘first contact the tax authorities’.*
(7) Listening to one’s own feelings. Respondents mention they should have listened to their gut feelings. For example: *‘listening to your inner feelings’*.
(8) Not listening to one’s own feelings. Some respondents, however, mention they should *not* have listened to feelings they had. For example: *‘if I were not so greedy’*.
(9) A third party should have done something. Some respondents mention that someone else (not the fraudster) should have done something to prevent the fraud. For example: *‘better inspection by Marktplaats’ (Marktplaats is an online trading platform; a Dutch version of eBay).*
(10) Rules and principles. In contrast to following one’s gut feelings, other respondents mentioned that they should have used safety rules and/or principles, which was this was worded as ‘never do …’ or ‘always …’. This includes using safer payment or trading methods (like only paying after receiving a product/service, or not conducting a transaction digitally but physically). Examples are: ‘*stronger control from [the online trading platform] and sharper from me. Do not pay immediately’, ‘do not download/share files via torrent. Better protection by torrent’*, and *‘first product then payments’*.
(11) Something else. Various answers were given that were rarer and/or not easy to classify. For instance, respondents mentioned *‘honest, well-paid employees at [online shop]’, ‘If more people know* [about them], *scams can be prevented’,* ‘*Better information about this scam’* and *‘if these persons are noticed earlier’.*

Again, codes for these possible strategies were not mutually exclusive. If respondents mentioned multiple possible strategies, multiple codes were assigned.

#### 2.3.3. Statistical analysis

The statistical analysis was based on unweighted data, with the exception of the presentation of the prevalence data. Chi-square tests and Pearson correlations were computed to analyze the relationships between variables; a Fisher’s exact test was used when more than 20% of the cells had expected cell counts lower than 5.

In the cross-tabular analysis of attempts strategies mentioned less than 25 times were not included to avoid focusing on details. In the cross-tabular analysis of victimization, this would have left almost nothing to analyze, therefore strategies that occurred less than 10 times were not included.

In a second step of the analysis, strategies of attempts were combined, for parsimony as well as for theoretical reasons, based on Street (2015)‘s ALIED framework, as well as the work of Levine and Daiku (2019), and Masip Pallejá et al. (2021) (see section 1).

A ‘combined knowledge’ strategy was created that consisted of four strategies described above: the first two strategies were (1) fraud knowledge and (2) using rules and principles, both of which could be regarded as ‘context-general information’. Both knowledge and rules and principles are ‘context-general information’, as described by Levine and Daiku (2019), Masip Pallejá et al. (2021), and by Street (2015). Two additional strategies, (3) spotting mistakes and (4) personal knowledge, were also included in this combined knowledge variable. Noticing mistakes as well as relying on personal knowledge could both be conceived as ‘individuating information’, or specific knowledge, in line with Street (2015)‘s theory of Adaptive Decision Strategies in Lie Detection (ALIED), as described above (see also Masip Pallejá et al., 2021).

The second combined strategy was ‘Verification of information’ which was the combination of: contact with others, independent information seeking, contact with the fraudster, contact with bank or credit card company, contact with police, contact with online shops & trading, and other preventive strategies. When respondents used knowledge as a strategy, other strategies were recoded as ‘not used’ in order to obtain a clear separation between respondents by strategy used. This was necessary as respondents could mention several strategies. All variables were coded as ‘strategy not mentioned’ versus ‘strategy mentioned’.

## 3. Results

### 3.1. Prevalence of fraud

By far the greatest part of the most important fraud incidents (68.9%) and fraud attempts (74.9%) took place online (percentages weighted to the Dutch population); 17.8% (frauds) and 7.2% (attempts) took place both offline and online and 13.2% (frauds) and 18.2% (attempts) took place completely offline. Although there were some differences between strategies by types of fraud, in all cases offline fraud constituted a minority of all cases.

Table 2 shows the victimization rate for the entire, representative sample. Online shopping fraud was the most common form of fraud: 10.5% in 2020. Six types of incidents had victimization percentages between 1 and 2% for 2020: identity fraud: 1.6%, friend-in-need fraud: 1.6%, charity fraud: 1.5%, investment fraud: 1.4%, phishing: 1.3% and lastly debt fraud: 1.1%. Finally, four types of incidents were reported by slightly less than 1% of the respondents in 2020: spoofing: 0.9%, price fraud: 0.9%, dating fraud: 0.9%, and finally job fraud: 0.2%. Another type of fraud was mentioned by 0.9% of the respondents. Attempted frauds were more common and did not entirely follow the same order of prevalence as the actual frauds. The most common attempts mentioned by respondents were phishing: 18.8%, online shopping fraud: 17.3%, spoofing: 14.5% and friend-in-need fraud: 12.9%. Other attempts occurred less often (Table 2).

**Table 2:** Prevalence of fraud victimization and attempts in the fraud victimization survey (percent as weighted to the Dutch population; *N* as in sample).

	Victim weighted %	*N*	Attempt* weighted %	*N*
Any fraud	15,7	424	41.7	1,203
Purchase fraud	10.5	282	17.3	475
Friend-in-need fraud (including ‘WhatsApp fraud’)	1.6	44	12.9	387
Identity fraud	1.6	45	5.2	155
Charity fraud	1.5	39	6.5	183
Investment fraud	1.4	42	8.5	258
Phishing	1.3	35	18.8	558
Debt fraud	1.1	29	9.7	286
Prize fraud	0.9	25	9.4	287
Dating fraud	0.9	20	2.4	61
Spoofing (including ‘helpdesk fraud’)	0.9	27	14.5	430
Other types of fraud	0.9	22	2.5	70
Job fraud	0.2	7	1.3	42
*N*		2,864		2,864

*Attempts include the victims.

It should be noted that the percentage of victims was comprised in the prevalence of the near victims. In other words, the number of people who experienced an attempt included both failed and successful fraud attempt.

This means that, for instance, 5.2% of the respondents experienced an attempt of identity fraud, among which 1.6% actually became a victim.

Although 34.5% of the respondents who experienced an attempt or became a victim indicated that they had no contact with the fraudster, 22.6% reported contact *via* email; 18.4% through an online trading platform, 7.5% through social media, 6.8% *via* telephone, 5.6% *via* an App and 2.6% *via* a text message. Finally, 5.2% met the fraudster(s) at home.

### 3.2. Strategies of near victims to avoid falling for fraud

A total of 960 respondents mentioned that they were aware of a failed attempt to defraud them and answered additional questions on the most memorable failed fraud attempt. Specifically, they were asked to provide a description of the fraud attempt, why they did not pay or lose money, and what they noticed that was not right (only if they indicated that they had noticed that something was not right).

Only 2 respondents did not answer the question on why they did not pay or lose money and also did not answer the question on what they noticed that was not right; they were thus excluded from further analysis. This left a sample of 958 respondents, all of which described the fraud attempt and indicated why they thought they did not lose money, and 859 respondents who specified noticing something that was not right.

The distribution of the fraud categories of these 958 respondents answering questions was: phishing (322), followed by friend-in-need fraud (127), debt fraud (114), spoofing (98), prize fraud (93), investment fraud (76), purchase fraud (34), other types of fraud (31), charity fraud (25), identity fraud (22), dating fraud (12), and job fraud (4). After coding, it appeared that 24 respondents did not properly answer the question and were marked as missing, after which these respondents were excluded from further analysis. This led to a final sample size of 934. Below, we describe the strategies near victims used to avoid fraud victimization in more detail (see Table 3).

**Table 3:** Prevalence of preventive strategies mentioned to avoid falling for fraud in order of prevalence, in percent (*N* = 934).

Preventive strategies	%	*N*
Quickly recognized	14.9	139
Fraud knowledge	69.0	644
Mistakes	27.9	261
Distrust	26.1	244
Rules and principles	11.7	109
Personal knowledge	7.1	66
Contact with others	5.5	51
Seeking information	4.0	37
Other preventive strategies	3.1	29
Contact with the fraudster	2.9	27
Contact with Bank or credit card company	2.2	21
Wise by experience	1.6	15
Contact with Police	0.2	2
Contact with online shops and trading	0.1	1

Respondents could mention several strategies. About half, 52.1%, mentioned only one strategy, 35.8% mentioned two and 10.5% mentioned three strategies. 1.6% mentioned 4 or 5 strategies.

#### 3.2.1. Strategies based on knowledge of fraud

Four strategies focused on the respondent knowing and/or recognizing something.

##### 3.2.1.1. Fraud knowledge

By far the most common preventive strategy was fraud knowledge (69%). Some respondents mentioned already ‘phishing’ in the description of the attempt. Several respondents mentioned that they recognized the fraud immediately: ‘*I immediately thought something was wrong*’. Some respondents mentioned they knew procedures of banks, tax authorities or other organizations and mentioned a mismatch with what happened during the fraud, for example: *‘I just knew it wasn’t real because I know the bank would never do this’, ‘the bank does not request information by email’ or ‘a bank never requests details via SMS’*. Respondents also mentioned having been informed by the media: *‘this way of scamming was extensively [covered] in the news’.* Finally, respondents mentioned specific characteristics of fraud that helped them recognize the attempt: *‘*[the] *email address was not correct’ and ‘[it was] clearly phishing’*. It was notable that many respondents appeared to be quite confident of their analysis by describing the incident as *‘it was clearly fake’, ‘it was clearly phishing’.*

Interestingly, several respondents provided us with instructions and tips on how to avoid fraud. For instance, one respondent mentioned that you needed to hover your mouse to detect a suspicious link, one respondent communicated that a delivery time of 2 weeks is often an indication of online shopping fraud, and another stated that it was important not to start a telephone conversation because the attacker may record your voice.

##### 3.2.1.2. Spotting mistakes

The second most common preventive strategy was spotting mistakes (27.9%). Many types of mistakes were reported. For instance, respondents noted that certain facts were incorrect: they did not order a package, did not have debts, did not have children, or did not bank with the bank mentioned in the fraudsters’ stories. Examples were: *‘the work charged had not taken place and I had never ordered it’, ‘I knew I had not ordered anything’,* and *‘I knew about the location, the chance that something would be built there was non-existent*’. Accordingly, the attacker had no chance of success. Some respondents also mentioned that if something seemed too good to be true, it probably was not: ‘i*f something is too good to be true, it usually is not true’* and *‘way too high return [on investment]*’. This was often mentioned for investment fraud attempts.

##### 3.2.1.3. Rules and principles

Personal rules and principles were the fourth most frequently reported preventive strategy (11.7%). Respondents mentioned personal rules or principles about always being alert, about checking things such as e-mail addresses and links, and about never doing certain things. Examples were: *‘I will not respond to an English-speaking person I do not know’, ‘I never pay to strangers via e-mail, not at all to the bank’, ‘I do not trust something like that from abroad and with a lot of language/spelling mistakes beforehand’, ‘I’m quite suspicious of such messages I do not go into unknown matters’, ‘I am always alert’.*

With respect to investment fraud, a respondent mentioned *‘I had no faith in investing in this area’.* With respect to charity fraud a personal rule was: *‘Even if it were true, I would not donate for these kinds of things’.* Regarding friend-in-need fraud one respondent mentioned*: ‘Because I do not pay on requests for a loan by WhatsApp’* and regarding identity fraud: *‘*[I] *never pay if I’m not sure of what, I’m suspicious’*. These personal rules and principles help them to avoid falling for fraud.

##### 3.2.1.4. Personal knowledge and private context

Personal knowledge was reported by 7.1% of the respondents. They noticed inconsistencies that related to personal knowledge rather than factual mistakes. Examples were: *‘my daughter would never ask for money via an app’, ‘the style did not correspond to what this family member normally uses’, ‘if it had been one of the children, they would have placed [a message in] the family app [group]’, ‘my children would never approach me like that, via WhatsApp’, ‘my daughter would never ask me for money’, ‘my father would never do that’*. These respondents had such confidence in their knowledge of their personal relations that they were sure that the fraudsters’ stories were incorrect.

#### 3.2.2. Distrust

Another preventive strategy consisted of negative gut feelings (26.1%); for respondents there was something odd about the fraud that generated negative feelings which protected them from becoming a victim. Respondents most often mentioned ‘*not trusting*’ the message or the situation, without being precise about why. For instance, they say: *‘[it] was too uncertain’, ‘[it] was very suspicious’, ‘[it was] not [to be] trusted’, ‘because I did not trust it and doubted it’, ‘[I] guess it was fake’, ‘the person insisted so much and accordingly I had to respond quickly otherwise it would not go through’*, and *‘it did not feel right’.*

#### 3.2.3. Wise through experience

Several respondents (15 respondents, 1.6% of the total) mentioned that they gained knowledge of fraud through previous experience with it. For instance, they wrote *‘investing has led to a lot of damage in the past’*, *[I] recognized the [fraud] trick, [it] had happened before, and then I fell for it’*, *‘did not trust it from previous experience’*, and *‘because I almost did not get [the money] back last time’.*

#### 3.2.4. Verification of information

Several methods were mentioned to search for information.

##### 3.2.4.1. Contact with others

Contact with others was cited by 5.5% of the respondents. These respondents indicated that they consulted others about the fraud, or sometimes that others contacted them about the fraud. They for instance had contact with family, friends, colleagues, or specific organizations, except those coded under other strategies. Examples were: ‘*Checked by calling her*’ (friend-in-need fraud) and ‘*Inspection via the authority, after which it turned out that it was indeed phishing’*.

##### 3.2.4.2. Independent information seeking

Four percent of the respondents detected the fraud attempt because they independently searched for more information. Examples of answers that were given were *‘just google it and you’ll see it’s wrong‘, ‘[I] looked up the number, [it] turned out to be a scam’, ‘then I go [went on] to investigate and [I] found that this party is unreliable’, ‘I have verified [it] and found that this was a scam’, ‘I googled it and found the same texts on forums where those people were warned’*, and *‘after a short [bit of] googling, it was fully confirmed to me that it was phishing’.*

##### 3.2.4.3. Checking with the fraudster

Information was checked with the fraudster by 27 respondents (2.9% of total) after which the fraud attempt was detected. This happened for instance for friend-in-need or investment fraud where elaborate communication with the fraudster was necessary for the fraud to succeed. For instance, with friend-in-need fraud, respondents asked which relative was contacting them, or with debt fraud respondents asked the fraudster to send the relevant purchase agreement. Respondents reported *‘because it was wrong from the start, check question with wrong answer. The girlfriend’s name was wrong’* and *‘it was clear that it could not be trusted; she would not say her name, only: I am your daughter. So [then] you already know enough’*.

Besides these 27, two respondents played a little game with the fraudster, while they recognized the fraud, they replied as if they were going along with it for a short period: ‘*it was a game on my part, answered a spam email’* (charity fraud) and ‘[I] *asked the fraudster (to play the game) to call me back via a landline. [He] did not bother me anymore’* (friend-in-need fraud).

##### 3.2.4.4. Contact with the bank or credit card company

Only 2.2% of the respondents contacted their bank for more information and thereby avoided falling for fraud. The bank stopped a transaction in 7 cases and the credit card company stopped a transaction in one case. In one case the respondent called his bank, and, as a result, the bank stopped the transaction.

##### 3.2.4.5. Contact with police

Two respondents contacted the police for information which resulted in not performing any transaction, and another one mentioned the police as the reason for not paying. They mentioned: *‘On the advice of the police I ignored the invoice and I never heard anything about it again’,* and *‘I found out through the police that it was a scam’.*

##### 3.2.4.6. Contact with online shops

One near victim mentioned that a large online shop helped him to notice fraud: ‘*Because, on the advice of the employee, I changed my password for [my] e-mail account and bol.com [Dutch large online shop]. I was very upset that I opened the email’.*

##### 3.2.4.7. Other preventive strategies

Respondents mentioned various other ways that they avoided victimization (3.2%). For example: *‘*[I have] *insufficient experience to start investing’, ‘[I] timely adjusted the login codes’,* and *‘because I’m broke’.* In this category there were also some near misses; for three respondents avoiding victimization was a matter of luck rather than intent, with them writing*: ‘[the] link [did] not work’, ‘payment was not possible’,* and *‘[I] could not login’*.

#### 3.2.5. Special cases

*Quick recognition or action.* Some respondents, 14.9%, mentioned explicitly that they recognized the fraud immediately. They often used words like ‘*directly’* and *‘immediately’.* For example, respondents wrote: *‘because I directly did not trust it’, ‘[my] alarm bells went off immediately’, ‘I directly called her’, ‘I directly verified this’, ‘because the phishing element was directly clear’, ‘because I directly thought ‘this is fake’*, and *‘I immediately knew it was not right’.*

*Contacting the impersonated person or organization.* In total, 75 near victims contacted someone else about the fraud (coded as either ‘contact with others’, ‘contact with the bank or credit card company’, ‘contact with police’, or ‘contact with online shops’). Among those 75 near victims, 43 (4.6% of all near victims) contacted the person or organization that the fraudster was posing as and they verified with the concerned party if the fraudster’s story was true (e.g., if they received a phishing e-mail from supposedly their bank, they contacted their bank about it). The other respondents that had contact with someone else about the fraud attempt, discussed it with someone who was otherwise not directly involved.

#### 3.2.6. Co-occurrence of preventive strategies

To investigate whether strategies were interrelated Pearson correlations coefficients were computed (Table 4). There were only a few significant correlations, namely 15 out of 72, not counting ‘quick response’, which is not a strategy in itself. When they were statistically significant, they were usually below |0.20|. Also, most statistically significant correlations were negative: when respondents mentioned one strategy, they tended not to mention other strategies (with correlation ranging from *r* = −0.07 to *r* = −0.20). There were a few positive correlations. A notable significant positive correlation: respondents who contact others also mentioned relying on personal knowledge (*r* = 0.17). Recognizing fraud quickly was associated positively with relying on knowledge to recognize fraud (*r* = 0.12) and with having personal knowledge (*r* = 0.07) but negatively with distrust (*r* = −0.07).

**Table 4:** Intercorrelations of strategies used in attempts, by type of fraud, Pearson Correlation (*N* = 934).

	Fraud know-ledge	Mistakes	distrust	Rules and principles	Personal know-ledge	Contacting others	Seeking information	Some-thing else	Contact fraudster	Contact bank	Wise-experience	Contact police	Contact shop
Quick decision	0.12^**^	−0.01	−0.07^*^	−0.03	0.07^*^	0.01	−0.05	−0.06	0.04	−0.04	−0.01	−0.02	−0.01
Fraud knowledge		−0.20^**^	−0.20^**^	0.06	−0.17^**^	−0.14^**^	−0.05	−0.15^**^	−0.11^**^	−0.07^*^	−0.04	−0.02	−0.05
Mistakes			−0.14^**^	−0.02	−0.14^**^	−0.08^*^	0.04	−0.07^*^	−0.05	−0.08^*^	−0.04	0.02	0.05
Distrust				−0.04	0.02	0.06	0.03	−0.01	0.06	0.02	−0.06	0.03	−0.02
Rules and principles					−0.04	−0.04	0.03	−0.03	−0.04	−0.06	0.01	−0.02	−0.01
Personal knowledge						0.17^**^	−0.06	0.00	0.03	−0.04	−0.04	−0.01	−0.01
Contacting others							−0.05	−0.02	0.13^**^	0.00	0.04	−0.01	−0.01
Seeking information								0.00	0.03	−0.03	0.02	0.11^**^	−0.01
Something else									0.04	0.01	−0.02	−0.01	−0.01
Contact fraudster										−0.03	−0.02	−0.01	−0.01
Contact bank											−0.02	−0.01	0.00
Wise by experience												−0.01	0.00
Contact police													0.00
Contact shop													

* Correlation is significant at the 0.05 level (2-tailed).

** Correlation is significant at the 0.01 level (2-tailed).

#### 3.2.7. Prevalence of preventive strategies by fraud category

Results showed that there were clear differences between strategies used by type of fraud (Table 5). Various types of fraud led to different strategies. Fraud knowledge was almost always the most important strategy, with about 65% or higher, but it was used less often in investment fraud, debt fraud, friend-in-need, with percentages of 50% or less. Mistakes were noticed mostly in the case of debt fraud: 66.7% and less often with other types of fraud. Distrust was mentioned relatively often in the case of investment fraud (43.7%), purchase fraud (53.1%) and charity fraud (43.5%). Rules and principles were mentioned most often with investment fraud (18.3%), prize fraud (20.7), charity fraud (17.4%) and other fraud (22.2%). Personal knowledge was used in less than 3.7% of the cases but was used in 46.8% of the attempted frauds with friend-in-need. Contacting others occurred mostly with friend-in-need fraud (20.6%). Seeking information happened the most with debt fraud (8.8%) and other types of fraud (11.1%). Checking with the fraudster did not occur a lot, but mostly with charity fraud (13%). Other strategies were most often used with investment fraud (19.7%). There were no large differences in the extent to which respondents mentioned reacting quickly to the fraud attempt. However, a swift response is mentioned between 20.6 and 22.6% of the cases with prize fraud, friend-in-need fraud, and spoofing.

**Table 5:** Strategies used in attempts, by type of fraud, in percent, Chi-Square or Fisher exact test.

	Invest-ment fraud	Purchase fraud	Prize fraud	Debt fraud	Charity fraud	Other fraud	Friend-in-need fraud (including ‘WhatsApp fraud’)	Phishing	Spoofing	Chi-Square/Fisher exact test
Quick decision	7.0	3.1	20.7	14.0	4.3	7.4	20.6	14.7	22.6	Fisher’s exact: *p* = 0.013
Knowledge	49.3	40.6	82.6	64.9	69.6	44.4	47.6	83.8	75.3	Chi^2^ = 103.40 ***
Mistakes	15.5	6.3	20.7	66.7	4.3	33.3	18.3	26.9	32.3	Chi^2^ = 112.10***
Distrust	43.7	53.1	29.3	16.7	43.5	33.3	22.2	23.1	20.4	Chi^2^ = 37.61***
Rules and principles	18.3	9.4	20.7	5.3	17.4	22.2	7.9	10.6	12.9	Fisher’s exact: *p* = 0.006
Personal knowledge	1.4	0.0	1.1	0.9	0.0	3.7	46.8	0.3	2.2	Fisher’s exact: *p* < 0.001
Contacting others	0.0	6.3	0.0	10.5	0.0	0.0	20.6	1.9	2.2	Fisher’s exact: *p* < 0.001
Seeking information	7.0	6.3	2.2	8.8	0.0	11.1	0.8	2.5	5.4	Fisher’s exact: *p* = 0.007
Contact the fraudster	4.2	3.1	0.0	1.8	13.0	7.4	7.9	0.3	3.2	Fisher’s exact: *p* < 0.001
Other strategies	19.7	6.3	0.0	0.9	4.3	0.0	2.4	1.3	1.1	Fisher’s exact: *p* < 0.001
*N* ^a^	71	32	92	114	23	27	126	320	93	

Strategies mentioned less than 25 times are not included in the table.

a Numbers can vary slightly due to missing values.

*** Correlation is significant at the 0.001 level (2-tailed).

Fisher exact test.

For simplicity, most researchers adhere to the following: if ≤ 20% of expected cell counts are less than 5, then use the chi-square test; if > 20% of expected cell counts are less than 5, then use Fisher’s exact test. Both methods assume that the observations are independent.

### 3.3. Potential preventive strategies that victims could have used

Questions on the most important fraud were answered by 393 victims; 22% noticed, at the time, or in hindsight, that something wasn’t right which they could have taken more seriously; 77.9% did *not* notice anything that might indicate that they were scammed. Asked about who could have prevented the fraud, 38.2% of those respondents answered that no one could have prevented the fraud and 61.8% (243 respondents) believed that the fraud could have been prevented and described how. Together with the fraud description these answers were coded. The distribution of the fraud categories, that these 243 respondents answered questions for, was as follows: purchase fraud (140), followed by investment fraud (17), phishing (14), friend-in-need fraud (11), other types of fraud (11), charity fraud (10), identity fraud (10), dating fraud (9), debt fraud (8), spoofing (8), and prize fraud (5). After coding, it appeared that 21 respondents did not properly answer the questions and were marked as missing, after which these respondents were excluded from further analysis. This led to a final sample size of 222. Below, the potential preventive strategies are described in more detail (see Table 6).

**Table 6:** Potential future preventive strategies mentioned by victims to avoid falling for fraud, in order of prevalence, in percent frequencies (*N* = 222).

Potential future preventive strategies	%	*N*
Seeking information	25.2	56
Pay more attention	18.9	42
Third-party could have done something/is to blame	16.2	36
Safety rules/principles or safer way of paying/trading	14.4	32
Simply not doing it	10.8	24
Consult others	9	20
Think better	8.1	18
Distrust more	5.9	13
Listen to feelings	4.1	9
Consult the concerned person/organization	4.1	9
Something else	3.2	7
Not listening to feelings	0.9	2

#### 3.3.1. Independent information seeking

Fraud victims most commonly (25.2%) said that victimization could have been prevented by independently seeking more information. Respondents for example wrote *‘doing better research’, ‘I had not done research on the web shop where I ordered the product’, ‘by checking on the internet’, ‘asking for more information’, ‘doing more research about the app’,* and *‘read up on it better’.*

Reading reviews about a seller was also mentioned commonly: ‘*I first should have read reviews of the web shop’, ‘looking at the reviews better’, ‘reading the reviews about the seller’, ‘reading review[s] of the company’,* and *‘first properly checking the experiences of others with this website’.*

Seven respondents (3.2% of total) mentioned questioning the fraudster for more information as a way of independent information seeking to prevent fraud. For instance, they reported: *‘by asking better questions’, ‘by asking more questions’, ‘by asking for more information’, ‘by asking for proof’*, and *‘by asking the serial number’*.

#### 3.3.2. Paying more attention and being more alert

The second strategy (18.9%) that could have prevented victimization, according to the victims, was by paying more attention to information that was already present during the fraud. Respondents, for example, wrote *‘by paying more attention’, ‘[by] reading well’*, and *‘[by] being alert’*.

#### 3.3.3. Third party should have done something

A third option (16.2%) to prevent the fraud was through something a third party should have done. One respondent mentioned that there could have been ‘*better public education about this scam’*. Another respondent blamed his/her bank: *‘the reviews of the web shop were so bad that the bank could have known about this’*. Another respondent called for better inspection of platform users by the online trading platform *‘Marktplaats’* (the Dutch version of eBay). Yet another respondent indicated that PayPal could have blocked the transaction.

#### 3.3.4. Rules and principles

The fourth most common strategy (14.4%) mentioned by victims was by following safety rules and principles or making use of safer payment or trading methods. A respondent for example mentioned ‘*only picking up [purchased goods]*’ (rather than relying on the sender to send the purchased goods *via* postage). Another respondent proposed: *‘first [receiving] the product, then paying’*, and another respondent recommended *‘by not paying beforehand’*.

#### 3.3.5. Simply not doing it

The fifth strategy (10.8%) according to victims was by simply not taking the action that they took. Respondents declared this without specifying further. They wrote, for example: *‘I simply should not have fallen for it’, ‘[by] not clicking the link’, ‘[by] not responding’, ‘by not opening the mail’, ‘by not ordering there’*, and *‘[by] not doing it’*.

#### 3.3.6. Contact with others

Next, 9% of the victims reported they could have avoided the fraud by consulting others about the fraud. A respondent wrote: *‘[by] telling this to a friend who could have advised me to not do it’*. Another respondent wrote: *‘[by] discussing [it] with family before [making the] investment’*, and yet another noted: ‘*if I had discussed it with someone before transferring money’*.

Nine respondents (4.1% of the total) mentioned that they should have contacted the concerned person or organization (that the fraudster was posing as), to verify the fraudster’s story. A respondent declared about preventing friend-in-need fraud victimization: *‘[by] first seeking contact with my son [to verify] if this WhatsApp [message] was right’*. Another respondent wrote about preventing debt fraud: *‘[by] first seeking contact with the tax authorities.*

#### 3.3.7. Thinking better

Another strategy, mentioned by 8.1% of the victims, was by thinking better before the action they took. This included taking more time before doing something. Respondents wrote, for example: ‘by thinking better’, *‘[by] thinking logically’, ‘[by] using common sense’,* or *‘by taking more time to think’*. Three respondents (1.4% of the total) specifically mentioned they should have taken more time to think; one respondent (0.5% of the total) on the other hand indicated that they should have thought quicker (*‘thinking quicker’*).

#### 3.3.8. Distrusting more

The eighth most common way that victimization could have been prevented was by being more distrusting and/or less gullible. Respondents specified, for instance: ‘[by] *not trusting everyone’, ‘[by] being more distrusting’, ‘not trusting everything’, ‘not believing everything that someone else says’*, and *‘[by] not being gullible’*.

#### 3.3.9. (Not) listening to one’s feelings

The ninth and tenth most common strategies that could have prevented fraud victimization were by either listening to one’s feelings (3.6%) or conversely not listening to one’s feelings (0.9%). About listening to one’s gut feeling respondents reported, for example: ‘[by] *trusting my feeling’*, ‘[by] *listening to my inner feeling*’, ‘*[by] following my instinct and not ordering’* and ‘*Listen to my own feelings and not my girlfriend’*. About *not* listening to one’s feeling respondents wrote: ‘*If I were not so greedy’* and ‘[by] *not letting me be tempted to more money’*.

#### 3.3.10. Other potential preventive strategies

Finally, victims mentioned some other ways that fraud victimization could have been prevented (3.2%). One respondent (0.9%) for example mentioned he/she should not have listened to their friend, who convinced them to make a fraudulent investment.

#### 3.3.11. Co-occurrence of potential preventive strategies

Pearson correlation coefficients were computed for each strategy (Table 7). There were mainly negative correlations, with some being significant. As was the case with the near victims, mentioning one strategy led to a lower likelihood of mentioning another as well. Victims who mentioned *‘simply not doing’* mentioned significantly less often that they should pay more attention, that a third party was to blame or that they should have followed safety principles. Furthermore, victims who proposed to independently search for more information mentioned less often that they needed to consult others, that a third party was to blame or that they should follow safety principles. Victims who mentioned that they needed to have higher feelings of distrust had a relatively low likelihood of searching for information. Interestingly, those who mentioned that they needed to think better also mentioned that they should not listen to their feelings. This was the only positive correlation.

**Table 7:** Interrelationship between potential preventive strategies, mentioned by victims, Pearson correlation of codes (*N* = 222).

	Simply not doing it	Distrust more	Think better	Pay more attention	Independently seek information	Consult others	Listening to feeling	Not listening to feeling	Third-party could have done something/is to blame	Safety rules/principles or safer way of paying/trading
Simply not doing it		−0.03	−0.05	−0.17*	−0.2**	−0.06	−0.07	−0.03	−0.15*	−0.14*
Distrust more			0	−0.02	−0.14*	−0.01	−0.05	−0.02	−0.11	0.01
Think better				−0.06	−0.1	−0.04	−0.06	0.15*	−0.09	−0.07
Pay more attention					−0.07	−0.11	−0.1	−0.05	−0.21**	−0.17*
Independently seek information						−0.18**	−0.12	−0.06	−0.2**	−0.15*
Consult others							−0.06	−0.03	−0.14*	−0.13
Listening to feeling								−0.02	−0.03	−0.08
Not listening to feeling									−0.04	−0.04
Third-party could have done something/is to blame										−0.08
Safety rules/principles or safer way of paying/trading										

#x002A;Correlation is significant at the 0.05 level (2-tailed).

**Correlation is significant at the 0.01 level (2-tailed).

#### 3.3.12. Prevalence of potential preventive strategies by fraud category

The occurrence of the potential preventive strategies by the six most common fraud categories is presented in Table 8. Fisher’s exact tests (used instead of a Chi-square test because for each code more than 20% of the cells had less than 5 observations) indicated no significant relations between any of the possible potential preventive strategies and fraud category.

**Table 8:** Most common potential strategies mentioned by victims, with Fisher exact test, in percent.

	Investment fraud	Purchase fraud	Charity fraud	Friend-in-need fraud	phishing	Identity fraud	Fisher exact test
Simply not doing it	7.7	5.3	11.1	11.1	30.8	12.5	*p* = 0.26
Distrust more	15.4	4.5	0	22.2	0	0	*p* = 0.31
Think better	23.1	6.1	11.1	11.1	7.7	0	*p* = 0.13
Pay more attention	7.7	16.7	22.2	11.1	46.2	25	*p* = 0.70
Independently seek information	7.7	34.1	33.3	33.3	7.7	12.5	*p* = 0.43
Consult others	23.1	3.8	11.1	22.2	0	12.5	*p* = 0.34
Listening to feeling	7.7	5.3	11.1	0	0	0	*p* = 0.72
Not listening to feeling	7.7	0.8	0	0	0	0	*p* = 0.47
Third-party could have done something/is to blame	15.4	16.7	0	22.2	15.4	25	*p* = 0.40
Safety rules/principles or safer way of paying/trading	0	19.7	0	0	0	25	*p* = 0.88
*N*	17	140	10	11	14	10	

### 3.4. The combined strategies used by near victims and likelihood of victimization

An important issue is whether the strategies used by near victims helped to prevent victimization of one of the fraud types measured in the present study. To investigate this, four cross tables were created of the four strategies, namely the combined knowledge strategy, verification of information, distrust and wise by experience, with victimization in 2020, as was mentioned above (section 2.3.3).

The results showed that the strategies used by near victims had a very different impact on the likelihood of victimization. Please recall that the overall victimization percentage was 15.7% (Table 2). Among those who had experienced a fraud attempt and were analyzed because they provided complete information, 17.9% became a victim of fraud. Table 9 shows that, when knowledge was used as a strategy, the likelihood of victimization was 15.3%, but when it is not used, the likelihood of victimization was 35.6%. Consequently, using knowledge as a strategy decreases the probability of victimization by a factor of 0.43 (see ‘ratio’ column, Table 9).

**Table 9:** Respondents who experienced an attempt and who were victimized Chi-Square, in percent, namely the percentage of respondents that was victimized when a specific strategy was absent or present.

Became a victim in 2020	Strategies		Ratio	Pearson Chi-Square	df	Significance
	Absent	Present	Present/absent			
	Combined knowledge strategies^a^					
Victimized	35.6	15.3	0.4	28.9	1	<0.001
*N*	118	816				
	Distrust					
Victimized	16.7	26.5	1.6	6.6	1	0.010
*N*	821	113				
	Wise by experience					
Victimized	17.7	42.9	2.4	18.4	1	<0.11*
*N*	927	7				
	Verification of information^b^					
Victimized	16.9	27.0	1.6	5.5	1	0.019
*N*	845	89				

*Fisher exact test.

a Knowledge: sum of fraud knowledge, mistakes, rules and principles and personal knowledge. The new variable was dichotomized into ‘strategy not mentioned’ versus ‘strategy mentioned’.

b Verification of information: sum of contact with others, independent information seeking, other preventive strategies, contact with the fraudster, contact with bank or credit card company, contact with police, and contact with online shops and trading. The new variable was dichotomized into ‘strategy not mentioned’ versus ‘strategy mentioned’.

When respondents mentioned knowledge, other strategies were recoded to zero, to avoid double coding in the present table.

In contrast, all other strategies increased the likelihood of victimization. Thus, when distrust was the strategy of choice, the likelihood of victimization is 26.5%, and when it is not used, the likelihood of victimization decreased to 16.7%, accordingly, using distrust increased the likelihood of victimization by a factor 1.6. Among respondents who mentioned they were wise by experience, 42.9% became a fraud victim, instead of 17.7%, an increase by a factor 2.4. Finally, when respondents wanted to verify information, 27% were victimized, and when this strategy was not used, victimization decreased to 16.9%. Clearly, it seems that having fraud knowledge is the best option to avoid victimization.

## 4. Discussion and conclusion

The purpose of this study was to gain a better understanding of how fraud victimization may be prevented. To this end we analyzed the answers on open questions about the strategies used by near victims to resist a fraud attempt and what strategy victims in hindsight thought could have prevented their victimization. Similar to Park et al. (2002), Levine and Daiku (2019), Blair et al. (2010), Masip and Herrero (2015), Masip Pallejá et al. (2021), and Novotny et al. (2018) we explored how this was done ‘in real life’ in a national random sample of respondents. Below we summarize the main findings and examine whether these can be connected to concepts proposed in the literature.

Overall, 15.7% of the respondents were a victim of fraud and 41.7% encountered an attempt. The number of attempts is relatively high and suggests that many people will encounter an attempt to defraud them at least once in their lifetime and may become a victim of fraud. Almost all fraud took place online. Among the (near) victims, 65.5% had some form of contact with the fraudster, generally through online communication channels.

Despite evidence for the existence of a Truth-Bias (Bond and DePaulo, 2006; Burgoon and Levine, 2010; Street, 2015; Street et al., 2019; Armstrong et al., 2021; Masip Pallejá et al., 2021; Levine, 2022) the relatively high number of attempted fraud victims relative to the number of actual victims suggests that there are more failed attempts then ‘successful’ attempts in fraud. This underscores the importance of context (Burgoon and Buller, 2015; Street, 2015; Street et al., 2019; Masip Pallejá et al., 2021) and lends some credence to the statement that in situations where people encounter an attempted fraud they may tend towards a ‘lie-bias’, as was suggested by Street (2015).

### 4.1. Preventive strategies used by near victims to avoid falling for fraud

The main strategy of near victims to avoid victimization is fraud knowledge; for more than two third of the near victims, what they knew about fraud allowed them to detect the fraud attempt. They were often confident and quick in their decision-making. Even when a quick decision wasn’t mentioned, they were often clear-cut in their judgment.

Previous research presented contradictory results on the importance of knowledge to avoid victimization. Several quantitative surveys concluded that knowledge of online fraud, and (un)safe behavior online behavior was unrelated to fraud victimization (Holt et al., 2018; Leukfeldt et al., 2018; Van’t Hoff-De Goede et al., 2019). Lea et al. (2009) reported that victims, who have a great deal of field knowledge overestimate their abilities to make good decisions and accordingly, are relatively likely to fall for a scam in that field. For instance, victims of investment fraud had more knowledge in finance than non-victims and were relatively likely to fall for an investment scam. The present study however, focused on ‘fraud knowledge’, not on knowledge in one particular field.

In accordance with what has been stated above (see also section 1), it is necessary to underline that there are different forms of knowledge that we have encountered in the literature and in this study. Above we concluded that those who avoided victimization recognized the fraud as a scam and we described this as ‘fraud knowledge’. Other studies mentioned above (Holt et al., 2018; Leukfeldt et al., 2018; Van’t Hoff-De Goede et al., 2019) regarded knowledge of ICT security as ‘knowledge’. These studies operationalized knowledge as recognizing stronger versus weaker passwords, identifying malicious URLs, or being able to define what a ‘firewall ‘is. We call this ‘ICT knowledge’. Finally, a third form of knowledge, used by Lea et al. (2009), is ‘field knowledge’, i.e., having knowledge of a specific field, for example having knowledge of the financial world. Based on the literature and current research, only fraud knowledge is important for the prevention of victimization, as previous studies concluded that ICT knowledge or field knowledge do not help to prevent fraud victimization. Experimental studies, just as the present study, did find fraud knowledge to be relevant (Grazioli and Wang, 2001; Kritzinger and von Solms, 2010, 2013; Hong, 2012; Purkait, 2012; Acquisti et al., 2015; Steinmetz et al., 2021; Dixon et al., 2022). Research on the effectiveness of training showed that improving knowledge reduces victimization of online fraud (Purkait, 2012; Bullée and Junger, 2020a). These findings also fit with psychological research that has emphasized knowledge to detect deception in an offline environment, as was stated by Levine and Daiku (2019), Masip Pallejá et al. (2021), and Street (2015).

Besides knowledge, additional strategies were mentioned as well but at much lower rates (28% or less).

Some strategies were used relatively rarely: searching for facts, such as looking for information online; contacting others, or call one’s bank or the police was mentioned by 5.5% of the near victim or less. This contrasts with deception detection studies in real life and offline (Park et al., 2002; Blair et al., 2010; Masip and Herrero, 2015; Novotny et al., 2018; Levine and Daiku, 2019; Masip Pallejá et al., 2021) where the role of additional information was more important.

### 4.2. Timing of detection

Another difference between offline and online interactions is the time that is needed to detect the truth. Deception detection studies reported that lies are often discovered relatively late and well after the fact (Park et al., 2002; Blair et al., 2010; Masip and Herrero, 2015; Levine, 2019; Masip Pallejá et al., 2021). For instance, 39.7% of the individuals who were lied to discovered this more than a week later (Park et al., 2002).

This relatively late detection offline contrasts with how online users seem to react to online messages. Respondents who mentioned relying on knowledge, mistakes, or personal knowledge generally ‘just knew’ right away and did not have to look for any additional information. Similarly, rules and principles were a guideline right away.

Speed of reaction time may be one of the differences between offline and online behavior. Usually, online users tend to react very quickly to messages. The likelihood that a user, who clicks on a malicious link, does this in the first 60 s is about 30% (Brink, 2018). Between 60 and 90% click within 12 h on a link in a phishing email and no one falls for a phishing email after 24 h (Mihelič et al., 2019; Jampen et al., 2020). This is surprising to some extent, as one might argue that there usually is no need to react fast online to, for instance, a specific email. But in practice, online users tend to react rather fast.

Near victims who mentioned one strategy usually did not mention another strategy. This may be the result of our methodology: when replying, respondents apparently tended to focus on one strategy and not mention another. It may also occur because, once a fraud attempt is detected *via* one strategy, further evaluation *via* other strategies is not relevant or necessary.

Globally, the rank order in strategies was often similar across the various types of fraud, with knowledge usually being the most important strategy, and with mistakes and feelings of distrust following. But some types of fraud seem to give rise to specific preventive strategies:

- To avoid falling for investment fraud, near victims use knowledge less often but listen to their feelings of distrust and follow their own rules and principles relatively often. This seems plausible as online offers often cannot be checked easily or refer to future profits that are hard to verify.
- To detect debt fraud, near victims most often noticed mistakes. This also seems plausible, as debt fraud often refers to something the near victim can verify with their own information, such as due taxes, or a package they supposedly bought.
- Near victims of friend-in-need fraud make use of fraud knowledge less often; instead, they commonly use personal knowledge. This makes sense as the fraudster is often impersonating someone known to the victim and therefore the near victim disposes of first-hand knowledge on the person who is being impersonated. These near victims also contact others more frequently. It is common sense to verify the content of the message with that specific person.

These findings show that near victims use different types of knowledge depending on the specific fraud forms they encounter.

### 4.3. Potential preventive strategies that victims could have used

The most common strategy mentioned by victims, that could have prevented falling for the fraud, was seeking for more information, such as reading reviews. A variety of other strategies were also mentioned, such as being more alert, relying on a third party, following certain rules and principles, contacting others or being more suspicious. As was the case with the near victims, victims who mentioned one strategy mentioned other strategies less often. There were no clear differences between the various strategies per the type of fraud. In part this could be the result of the relatively low number of victims in some categories.

### 4.4. Comparison of near victims and victims

When comparing the answers of the near victims with the actual victims a number of things stand out. First, there seems to be a difference in the degree of confidence between both victims and near victims. Respondents experiencing attempts were relatively clear in their answers: they overwhelmingly mentioned the use of fraud knowledge, followed by spotting mistakes and by listening to their own feelings of distrust. Looking back at what might have helped to avoid victimization, about 40% of the actual victims thought nothing might have been done. Only about one-fifth mentioned that they had noticed beforehand or in hindsight, that something wasn’t right. When asked about preventive strategies, only about half could provide an answer. Second, victims’ answers were much less consistent and more spread out over the various categories. Third, there is a discrepancy between what helped near victims to avoid victimization and what the actual victims believed about how to prevent fraud. Victims proposed strategies such as seeking information, relying on a third-party to do something, simply not doing it, consulting others, distrusting more, or listening (or not) to feelings were actually associated with higher and not lower likelihood of victimization. In contrast, near victims hardly ever searched for information online because they had already recognized the fraud or were sufficiently on their guard. Accordingly, searching online was not necessary anymore. It is unclear if strategies proposed by victims such as ‘thinking better’ and ‘paying more attention’ would be helpful in the future. Only 14.4% of the victims proposed safety rules and principles or safer ways of paying/trading as a strategy to prevent fraud victimization, which appeared to help near victims to avoid victimization.

All this suggests that numerous victims still have trouble understanding what had happened and were somewhat at a loss. This matches with reports by Whittaker et al. (2022) who found that 44% of victims who were scammed and reported to Scamadviser [a fraud information and reporting website (see Scamadviser, 2023)] noticed the scam too late and 20% mentioned that they lacked knowledge. Whittaker et al. (2022) also reported that victims mostly used strategies that were not effective in identifying a scam. Razaq et al. (2021), similarly, emphasized victim’s vulnerabilities. They described how some near victims in Pakistan were so enthralled by the possibility of winning a big prize that they could not be persuaded by relevant others that they were about to fall for a scam and should not pay. Taken all together, this seems to imply that a relatively large group of victims has insufficient knowledge of fraud, were perplexed, and still, after the fact, they had not managed to build a strategy for themselves that may work in the future.

Our results have implications for fraud prevention. We relied to a large extent on the comparison of the results of the victims with the near victims, it was the contrast between the stories of near victims and victims that is key to understanding how to avoid fraud.

Today, there is an enormous amount of online information on online security and online fraud. Practically every bank, insurance company, government organization, and law enforcement website provides webpages devoted to warn users and provide tips and guidelines on how to stay safe online (Whittaker et al., 2022). Apparently, this is not enough to curb the rising trends of online fraud that were described above. A disadvantage of this system of providing information is that users have to actively search for it. But they probably do not do so often. Many studies stated that security is seldom a user’s first priority (Krol et al., 2012, Acar et al., 2016, Junger et al., 2017). Accordingly, when online, users are probably busy with other activities.

Therefore, we believe that the public should be proactively informed about fraud much more than is the case today. Instead of an information search process where users have to initiate a search, proactive information aims to identify users current information needs. Proactive information is necessary as those who become a victim obviously do not recognize the fraud and therefore generally do not start searching for additional information. Therefore, providing online information and hoping that users will find it, is not sufficient. This implies that public and private organizations should actively reach out to the general public, as well as to specific groups, such as students and the elderly. This could be done through media campaigns, in newspapers or on television or other media, that provide general as well as specific information about fraud with the aim to increase knowledge of the general public and propose guiding principles.

Besides, courses on online safety as well as on online fraud should be provided to students in educational institutions, to employees and to the elderly. Furthermore, a specific high-risk group are the first-time victims, as the level of repeated victimization is relatively high. Junger et al. (2022) reported that 40.2% of the fraud victims in the present sample are victimized more than once, in contrast with the overall victimization rate of 15.8%, a common finding for online and offline victimization (Farrell and Pease, 2018; Moneva et al., 2021). They could be reached after they reported their victimization, to the police, or to their bank or any other (victim) organization. In addition, information could be provided about how to act or where to find additional tips or tools, such as websites that check links for online users or where to find free anti-phishing training.

Implementing preventive policies, however, is easier said than done. While some were rather negative about teaching the public (Bada et al., 2015), research showed that there are effective interventions (Purkait, 2012; Purkait et al., 2014; Bullée and Junger, 2020a) that prevent falling for online fraud. The effectiveness of large public media campaigns has not been evaluated, as far as the present authors are aware of.

It is likely that online users will remain vulnerable to online fraud in the coming years. We believe the most important task for researchers is to continue to develop interventions to prevent online fraud and test them, as well as connect to policymakers and test new policies. At present, our professional contacts with policymakers suggest that many public campaigns are not very effective. Accordingly, the effectiveness of these campaigns needs to be measured and improved. It is important to verify whether they reached the right target group, or the majority of the public, if they were understood and if they managed to have an impact on fraud victimization.

Bullée and Junger (2020a) listed several problems in the field of online fraud prevention. As mentioned above, it is difficult to find cross-situational indicators of fraud (Burgoon and Levine, 2010; Purkait, 2012; Button et al., 2014; Burgoon and Buller, 2015). Fraud comes in countless varieties, and different types of fraud have different modus operandi. Fraud knowledge generally implies some familiarity with the specific modus operandi of a specific type of fraud. An important issue is to what extent one can warn the public against fraud in general or whether specific information is necessary that warns against each modus operandi. Despite many issues to solve, the present findings strongly point to the need to setting up larger efforts to proactively inform the public about fraud, as suggested by Whittaker et al. (2022).

Our study has a number of limitations. First, although we asked questions about victimization of many types of fraud, and added a question about ‘other types of fraud’, one can never be certain that all fraud victimization was measured. Second, due to the skewed nature of fraud victimization, some types of fraud, such as job fraud, had very low prevalence. Third, we cannot be certain that the fraud that targeted the near victims was similar to the type of fraud that targeted the actual victims. Although we could control for the type of fraud, this analysis may need more precision.

Despite these limitations, our study provides valuable insights into how near victims can avoid victimization and how actual victims believe they might have prevented their victimization. To the best of our knowledge, this is the first overview of preventive strategies used by near victims and actual victims of fraud based on a representative sample and making use of victims’ own accounts. Our main findings showed that near victims can avoid victimization when they already have knowledge of fraud and consequently, they recognize it when they see it. Our main suggestion for policymakers is to organize broad information campaigns to inform the public. Most victims do not visit websites for more information or consult others at the time they are confronted with a fraud attempt.

## Data availability statement

The datasets presented in this study can be found in online repositories. The names of the repository/repositories and accession number(s) can be found at: https://www.centerdata.nl/.

## Ethics statement

The studies involving human participants were reviewed and approved by BMS Ethics Committee /domain Humanities and social sciences (HSS) of the University of Twente. Written informed consent for participation was not required for this study in accordance with the national legislation and the institutional requirements.

## Author contributions

MJ contributed to the conception and design of the study, the acquisition, the analysis, and the interpretation of the data, and main writer of the manuscript. LK participated in the initial design of the study, the pre-processing of the data, the data analysis, and the writing corrections of the various manuscript versions. MJ, LK, and PH developed both codebooks and worked on the coding of the victims-and near victims’ answers. PH critically reviewed the article. BV contributed to the design of the study, advised and provided feedback on pre-processing of the data and the data analysis, final manuscript. All authors approved the final version of the manuscript and are accountable for all aspects of the work with regard to questions related to the accuracy or integrity of any part of the work that are appropriately investigated and resolved.

## Funding

The research was funded by Stichting Achmea Slachtoffer en Samenleving (SASS) (Achmea Victim and Society Foundation), as well as International Card Services (ICS), the National Police and the Dutch Banking Association (NVB).

## Conflict of interest

The authors declare that the research was conducted in the absence of any commercial or financial relationships that could be construed as a potential conflict of interest.

## Publisher’s note

All claims expressed in this article are solely those of the authors and do not necessarily represent those of their affiliated organizations, or those of the publisher, the editors and the reviewers. Any product that may be evaluated in this article, or claim that may be made by its manufacturer, is not guaranteed or endorsed by the publisher.

## Footnotes

^1^A five-year screening question helps recall and was used to allow respondents to report relatively recent incidents but, at the same time, avoid forward telescoping problems, that is, help respondents not ‘push’ older incidents forward and bring them into the reporting period (Tourangeau and McNeeley, 2003). Measuring crime and crime victimization: Methodological issues. In: Pepper, J. V., Petrie, C. V. (Eds.) Measurement problems in criminal research: Workshop summary. Washington, DC: The National Academies Press, (Reep, 2017). Fraude met online handel. Antwoorden uit de Veiligheidsmonitor vergeleken met het politieregister (Online trading fraud. Information from the Security Monitor compared with the Police Register). Methodologie paper. Den Haag, NL: Statistics Netherlands.

^2^The codebooks can be obtained from the second author.
