# Supplementary material for: In their own words: deception detection by victims and near victims of fraud
Source: Front Psychol. 2023 May 12;14:1135369. doi: 10.3389/fpsyg.2023.1135369 (PMC10213419; doi:10.3389/fpsyg.2023.1135369)
Supplement: Supplementary file 1 [file Data_Sheet_1.docx]

**Online appendix**

1. Kappa and agreement between coders

Table 1: Agreement between coders after discussion to control for differences in views, percent agreement, Kappa, and significant test, N= 958

| Attempts | Both | LK | MJ | None | Kappa | p |
| --- | --- | --- | --- | --- | --- | --- |
| Quick | 99.3 |  | 0.7 |  | 1.00 | *** |
| 1) Fraud knowledge | 97.8 |  | 2.2 |  | 0.97 | *** |
| 2) Feeling-Distrust | 99.6 |  | 0.4 |  | 1.00 | *** |
| 3) Rules and principles | 93.2 | 6.8 |  |  | 0.96 | *** |
| 4) Information seeking | 100 |  |  |  | 1.00 | *** |
| 5) Mistakes | 93.2 | 6.5 | 0.4 |  | 0.95 | *** |
| 6) PERSONAL knowledge | 95.5 | 1.5 | 3 |  | 0.98 | *** |
| 7) Contact with the bank of credit-card company | 100 |  |  |  | 1.00 | *** |
| 8) Contact with online shops | 100 |  |  |  | 1.00 | *** |
| 9) Contact with others | 100 |  |  |  | 1.00 | *** |
| 10) Wise through experience | 100 |  |  |  | 1.00 | *** |
| 11) Contact with Police. | 100 |  |  |  | 1.00 | *** |
| 12) Check with attacker | 100 |  |  |  | 1.00 | *** |
| 13) Something else | 96.6 |  | 3.4 |  | 0.98 | *** |
| Missing | 82.8 | 17.2 |  |  | 0.90 | *** |

Table 2: Agreement between coders after discussion to control for differences in views, percent agreement, Kappa, and significant test, N= 243

| **Actual victims** | Both | LK | MJ | None | Kappa | p |
| --- | --- | --- | --- | --- | --- | --- |
| 0 Simply don't go along, just don't trust | 15.2 | - | - | 84.8 | 1 | *** |
| 1 Pay more attention, think, take more time | 23.9 | - | - | 76.1 | 1 | *** |
| 2 Search yourself extra info from third sources or request extra info from fraudsters | 42.1 | - | - | 57.9 | 1 | *** |
| 3 Consult others (not the involved person/organization) | 4.5 | - | - | 95.5 | 1 | *** |
| 4 Consult the person/organisation | 3.7 | - | - | 96.3 | 1 | *** |
| 5 listening to feelings | 3.3 | - | - | 96.7 | 1 | *** |
| 6 Not listening to feelings | 0.8 | - | - | 99.2 | 1 | *** |
| 7 Third party should have done something/is part guilty | 14.8 | - | - | 85.2 | 1 | *** |
| 8 Security rules, principles, other 'secure' methods of payment/trading | 13.2 | - | - | 86.8 | 1 | *** |
| 9 Other remark | 2.9 | - | - | 97.1 | 1 | *** |
| 99 Missing | 8.6 | - | - | 91.4 | 1 | *** |

2. Graphs based on table 5: Strategies used in attempts, by type of fraud, in percent.

Figure 1: Quick decision as a reaction to an attempted fraud, by type of fraud, in percent (see also text, table 5)

Figure 2: Fraud knowledge during an attempted fraud, by type of fraud, fraud, by type of fraud, in percent (see also text, table 5)

Figure 3: Noticing mistakes during an attempted fraud, by type of fraud, fraud, by type of fraud, in percent (see also text, table 5)

Figure 4: Feelings of distrust during an attempted fraud, by type of fraud, fraud, by type of fraud, in percent (see also text, table 5)

Figure 5: Using rules and principles during an attempted fraud, by type of fraud, fraud, by type of fraud, in percent (see also text, table 5)

Figure 6: Using personal knowledge during an attempted fraud, by type of fraud, fraud, by type of fraud, in percent (see also text, table 5)

Figure 7: Contacting others during an attempted fraud, by type of fraud, fraud, by type of fraud, in percent (see also text, table 5)

Figure 8: Independent information seeking during an attempted fraud, by type of fraud, fraud, by type of fraud, in percent (see also text, table 5)

Figure 9: Contacting the fraudster during an attempted fraud, by type of fraud, fraud, by type of fraud, in percent (see also text, table 5)

Figure 10: Using others strategies during an attempted fraud, by type of fraud, fraud, by type of fraud, in percent (see also text, table 5)
